# Protocol for inducing branching morphogenesis in human cholangiocyte and cholangiocarcinoma organoids

**DOI:** 10.1016/j.xpro.2023.102431

**Published:** 2023-07-09

**Authors:** Kimberley Ober, Floris J.M. Roos, Gilles S. van Tienderen, Kübra Köten, Annelot Klaassen, Wunan Mi, Luc J.W. van der Laan, Monique M.A. Verstegen

**Affiliations:** 1 Department of Surgery, Erasmus MC Transplant Institute, University Medical Center Rotterdam, Rotterdam, the Netherlands; 2 Wellcome-MRC Cambridge Stem Cell Institute, University of Cambridge, Cambridge, UK; 3 Department of Surgery, University of Cambridge and NIHR Biomedical Research Centre, Cambridge, UK; 4 Department of Surgery, Cambridge University Hospitals NHS Foundation Trust, Cambridge, UK

**Keywords:** Cancer, Cell Biology, Developmental biology, Organoids

## Abstract

Bile ducts are essential for bile transport and consist of complex branching tubular networks. Human patient-derived cholangiocyte develops a cystic rather than branching duct morphology. Here, we present a protocol to establish branching morphogenesis in cholangiocyte and cholangiocarcinoma organoids. We describe steps for the initiation, maintenance, and expansion of intrahepatic cholangiocyte organoids branching morphology. This protocol enables the study of organ-specific and mesenchymal-independent branching morphogenesis and provides an improved model to study biliary function and diseases.

For complete details on the use and execution of this protocol, please refer to Roos et al. (2022).^1^

## Highlights

• Protocol to induce tip-driven branching morphogenesis in human adult liver-derived ICO
• BRCO better represent the intrahepatic bile duct architecture *in vitro*
• BRCO can be expanded long term *in vitro*, while maintaining branching morphology
• Branching morphogenesis in BRCCAO is irregular and better represents the primary tumor

## Abstract (editor highlights)

Publisher’s note: Undertaking any experimental protocol requires adherence to local institutional guidelines for laboratory safety and ethics.

## Graphical abstract

**Figure:**
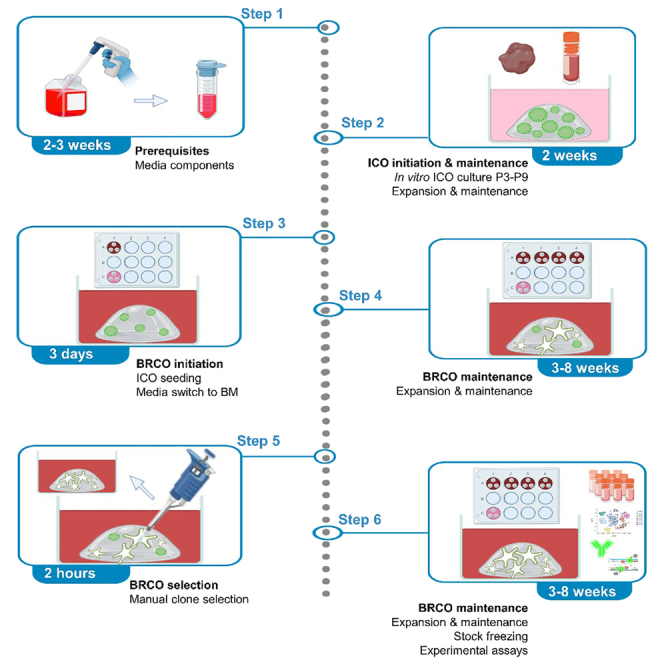

## Before you begin

This protocol describes the induction of branching morphology in human adult intrahepatic cholangiocyte organoids (ICO) and cholangiocarcinoma organoids (CCAO).^2^ These branching organoids (BRCO) grow into functional tubular structures resembling the biliary tree *in vivo*. In addition to healthy tissue-derived BRCO, branching morphologies could be induced in CCAO^3^ and polycystic liver disease organoids (PCLDO), providing a better model to study disease progression and novel treatment options *ex vivo*. Important to mention is the unsuccessful branching of fetal liver-derived organoids and extrahepatic cholangiocyte organoids, using this protocol.^1^

Although both liver tissue-derived and cryopreserved ICO present branching abilities, important criteria should be taken into consideration. First, successful BRCO can only be initiated in already established ICO (in culture for at least three passages), and not directly from liver biopsies. In addition, approximately 30% of ICO cultures will not show any branching structures upon initiation, even when correct procedure handling has been applied. Although extensive studies have been performed to elucidate this phenomenon in lack of branching morphology, until this date no specific underlying patient characteristics or mechanistic pathways have been identified. An extensive discussion and analysis of BRCO initiation and its use in biliary research, has been described by Roos et al*.*^1^

We hereby provide a detailed step-by-step protocol for the initiation, maintenance, and expansion of branching morphology in ICO. This protocol includes important technicalities that increase the chance of successful branching initiation.

**CRITICAL:** During *in vitro* branching initiation of ICO and expansion of the cultures, Biosafety level 1 regulations should be applied, according to standard aseptic techniques.

**CRITICAL:** To prevent any contamination of the organoid cultures, all procedures should be performed in a laminar flow cabinet using sterile reagents, media and disposables.

**CRITICAL:** All cell and organoid cultures should be maintained within a humidified cell culture incubator at 37°C with 5% CO_2_.

**CRITICAL:** All ICO and BRCO cultures should be maintained using suspension well-plates (ranging from 48-well to 12-well plates).

### Institutional permissions

ICO were initiated from healthy donor liver biopsies obtained during liver transplant procedures performed at the Erasmus MC, Rotterdam, the Netherlands. CCAO were initiated from tumors collected during surgical resection procedures for curative intent. All patients signed an informed consent to approve the use of the biopsies for research. The Erasmus MC Medical Ethical Committee approved the use of liver and tumor biopsies for research (MEC-2014-060 and MEC 2012-090, respectively).

### Preparation of required (conditioned) media components and stock solutions

**Timing: 3–4 weeks**

1. All commercially available components and (conditioned) media should be aliquoted and applied in the correct concentrations before (BR)ICO cultures are initiated, according to the manufacturer’s recommendations (see materials and equipment for details).
2. Generate conditioned medium (CM) for organoid culture by culturing stably transfected L-Wnt-3a, 293T-HA-Rspo1-Fc and Noggin HEK293 cell lines, for the production of Wnt-3a, R-Spondin and Noggin.^1^^,^^2^^,^^4^^,^^5^
  a. Twice a week, split or harvest the attached monolayer of cells upon 90%–100% confluency when log phase growth expansion is observed, not exceeding >10% debris or detached cells.
    i. Harvest medium by transferring into a 50 mL Falcon tube.
    ii. Centrifuge the collected media at 453 *g* for 5 min at 4°C.
    iii. Filter the supernatant through a 0.22 μm filter to avoid transfer of cells and debris.
    iv. Aliquot in 35 mL and store at −20°C (R-Spondin, Noggin) or 4°C (Wnt-3a) until further use.
      ***Note:*** Pool multiple batches to minimize variation in efficiency levels per batch round.
  b. Validate the quality and concentration of the harvested condition medium by applying ELISA (R-Spondin, Noggin) and the FOP-TOP assay (Wnt-3a), according to published instructions.^3^^,^^6^
  c. Every 3 months, check the cell lines for mycoplasma infection or other contaminations.
    ***Note:*** Basic culture experience and skills are preferential in order to plan and organize the collection of CM. This is especially critical for the timing of the first batch collection.
    ***Note:*** After cell line thawing and culture initiation, it is strongly recommended to let the HEK293T cells recover for at least two passages before the first batch of CM is harvested. Especially Noggin HEK293 can present itself as a poorly adhesive line directly after thawing, make sure to handle the cells gently upon washing.
    ***Note:*** Do not use CM-producing cell lines from which the media is harvested for further culture or seeding of new CM-producing batch, since these cells have been exhausted. Maintain a back-bone of the CM-producing cell line in culture, that can be used for seeding a fresh CM-producing badge for media harvest.
    ***Note:*** All three CM-producing cell lines can be long-term cultured until at least passage 80. It is recommended to perform (STR) genotyping when cell line exceeds passage 50, to ensure a representative cell line.
    ***Note:*** R-Spondin and Noggin CM can be stored long-term for at least one year in aliquots of 35 mL at −20°C, while R-Spondin and Noggin maintaining stable activity, including endurance of multiple freeze-thaw cycles. Additionally, 35 mL aliquoted Wnt-3a only remains stable for 3 months at 4°C.
    ***Note:*** Apply the CM according to the appropriate concentrations as mentioned in the culture protocol. If any divergent concentrations are measured during validation experiments, the amount added to the media should be amended accordingly.
3. Prepare the required organoid culture medium and filter through a 0.22 μm filter before use.

***Note:*** Organoid culture media can be stored for 1 week at 4°C, after which fresh organoid culture medium should be prepared.

***Note:*** All culture media added to the ICO, SEM, EM or BM, should be pre-warmed to 37°C before addition to the culture wells and BME domes. Upon cold media addition, the BME domes will disintegrate into a more liquid state while losing its ICO supportive matrix capabilities, and dispatches from the well bottom.

### ICO initiation and maintenance

**Timing: 3–4 weeks**

4. Either initiate ICO cultures from (cryopreserved) liver tissue biopsies or thaw cryopreserved, already established ICO.^1^^,^^2^^,^^4^^,^^7^
  a. Initiate ICO from at least 3 mm^3^ tissue biopsies, either healthy donor or patient derived materials, by 2.5 mg/mL collagenase IA (in 4 mL EBSS) digestion for 30 min at 37°C onto a rocking platform at 80 cycles/min.
    i. Following digestion, add 24 mL EBSS and filter the solution through a 100 μm filter to hamper the inclusion of residual debris.
    ii. Centrifuge at 453 *g* for 5 min at 4°C and wash the obtained pellet with 10 mL ADV+++ to remove residual collagenase IA.
    iii. Obtain the ICO by centrifugation at 453 *g* for 5 min at 4°C.
    iv. Resuspend the acquired pellet in 100% Basement Membrane Extract (BME, Cultrex or a similar type of hydrogel such as Matrigel-Corning) with the appropriate volume to seed 1–3 wells of a 48-well plate with one 25 μL dome.
      ***Note:*** Organoid culture experience and skills are preferential in order to determine the correct number of wells and the size of the suspension well plate for initiation seeding. The applied number of wells is based on pellet (and debris) size.
    v. Solidify the seeded domes by incubating at 21°C–25°C for 3 min.
      ***Note:*** Prevent any movement of the plate during the three-minute 21°C–25°C incubation for the solidification of the BME domes, thereby preventing a shift and destruction of the BME dome.
    vi. Flip the plate upside down in one smooth motion.
      ***Note:*** Flip the plate upside down in a smooth motion to ensure the distribution of the ICO through the complete BME dome. Otherwise, the ICO will sink towards the well-bottom within the BME dome, thereby limiting environmental growth space in 3D and potentially promoting well-bottom adhesion of the ICO towards a 2D outgrowth.
    vii. Incubate 45 min at 37°C.
    viii. Reverse the plate and add 250 μL Start-up Expansion Medium (SEM) to each seeded well of a 48-well plate.
    ix. Refresh SEM every 2–3 days for 5–7 days, by removing the old media and adding fresh 37°C pre-warmed SEM.
      ***Note:*** Directly after organoid initiation, only a few (+/- 10) small (Ø 10 μm) cell clumps and residual cellular debris can be observed, using a bright field microscope (Figure 1A; black squared box with zoom in section). These small cell clumps could grow out into small (Ø 10–100 μm) organoid structures after 5–7 days of culture (Figure 1B; black squared box with zoom in section).
      **Figure 1:**
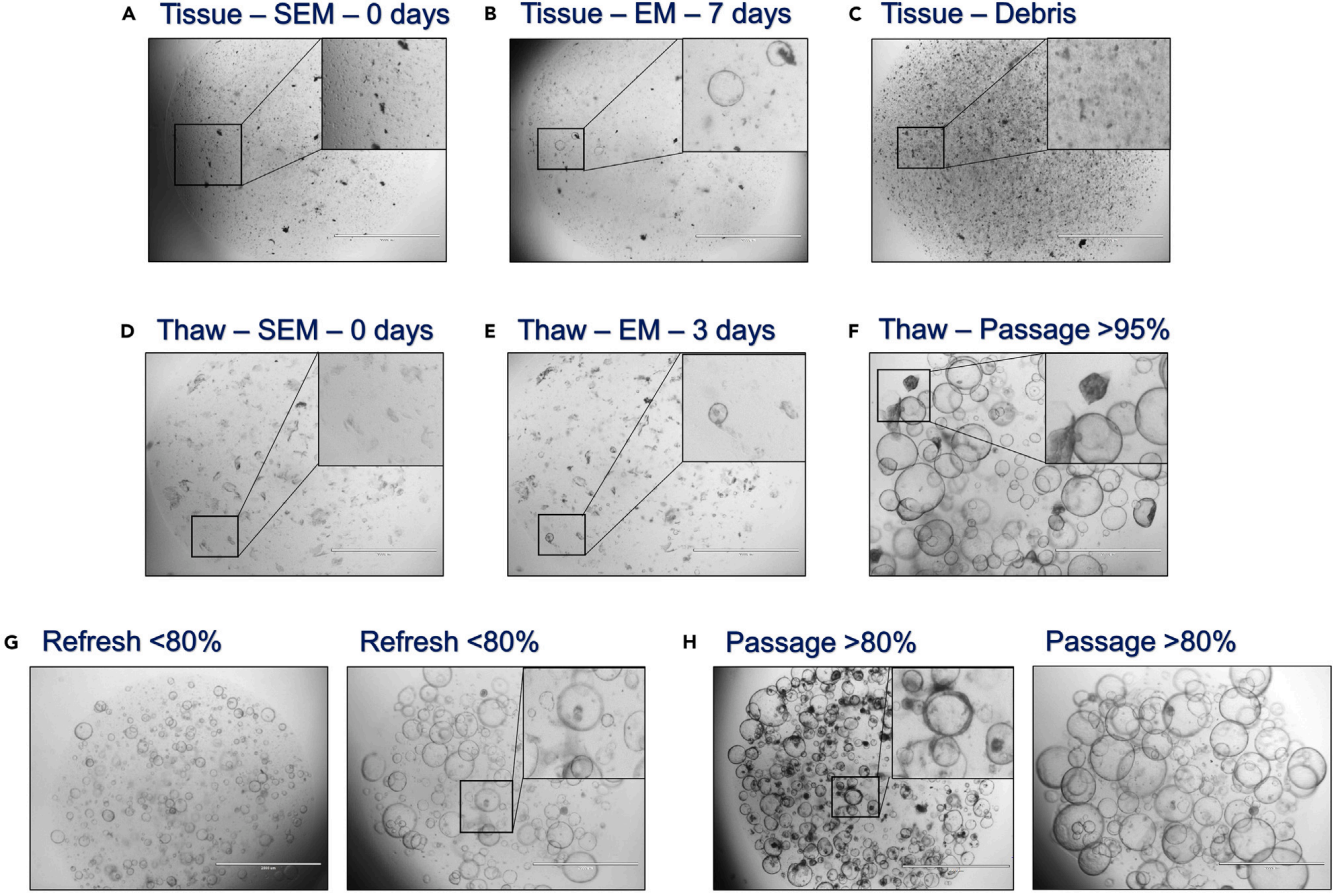
Bright field microscopic images of ICO culture maintenance after tissue or cryopreserved organoid culture initiation (A) Immediately after tissue-derived ICO initiation and SEM addition, showing residual cellular debris and only small potential ICO structures (Ø 5–10 μm; zoom in black squared box). (B) While residual cellular debris is still present, the SEM media is switched for EM after day 3 of initiation, showing ICO structures arising (Ø 10–100 μm; zoom in black squared box). (C) The maximum density of the residual cellular debris level upon tissue-derived ICO initiation, which will still result in viable ICO culture (zoom in black squared box). (D) ICO initiation from previously established cryopreserved ICO with an overall 50% debris density upon thawing. Only few small potential ICO structures are visible (Ø 5–10 μm; zoom in black squared box). (E) Upon multiple small ICO structures (Ø 10–100 μm; zoom in black squared box) the SEM media is switched for EM, after approximately 5 days. (F) 95% ICO density within the BME dome with a collapsed darkened ICO within the zoom in black squared box, indicating that passaging the culture is desired. (G) Examples of ICO cultures with densities <80% that only require media refreshment, no passaging yet, showing entrapped debris within an ICO in the zoom in black squared box. (H) Examples of ICO cultures with densities >80% that require passaging for culture expansion, in which the zoom in black squared box indicates the formation of thickened ICO borders within a dense ICO culture. (All images: 2× magnification; scale bar indicates 2,000 μm).
    x. Upon the formation of multiple small organoids (Ø 10–100 μm), after approximately 5–7 days, switch the SEM for Expansion Medium (EM), by removing the SEM and adding fresh 37°C pre-warmed EM.
      ***Note:*** Upon organoid initiation, only switch from SEM to EM when multiple small (Ø 10–100 μm) organoids are observed using a bright field microscope (Figure 1B; black squared box with zoom in section). Continue SEM refreshment up to 7 days after initiation, hereafter switch to EM even when no organoid structures are present.
    xi. Refresh the EM every 2–3 days by removing the old media and adding fresh 37°C pre-warmed EM.
    xii. Continue with the ICO maintenance and expansion protocol accordingly.
      ***Note:*** If no ICOs have initiated after 7 days of SEM and subsequent 7 days of EM incubation, deem the culture initiation as failed and discard the plate.
      ***Note:*** The amount of debris, such as other cells (erythrocytes) or cellular remains observed within the ICO culture, highly depends on the quality of the starting material and can differ greatly between different donors. More importantly, when the level of debris exceeds 90% BME occupation, it could greatly hamper viable ICO initiation (Figure 1C; black squared box with zoom in section). In addition, the stiffness of the BME could be adversely affected. It is strongly recommended to passage the fragile initiating ICO 1:2 to increase potential growth space and prevent the adverse effects of high debris levels (see step 5 for the procedure of ICO passaging and culture maintenance). Upon passaging, ICO should be harvested without any vigorous pipetting and only collecting the ICO once with centrifugation, followed by immediate BME seeding.
  b. Thaw cryopreserved established ICO cultures (preferably at passage 1–5), by gently swirling the vial at 37°C until a small frozen section is present.
    **CRITICAL:** Perform the procedure in a timely manner to avoid any harm to the ICO culture while promoting a fast and viable initiation.
    i. Add 9 mL ice-cold ADV+++ slow and dropwise to the thawed ICO, while swirling the tube upon buffer addition.
    ii. Collect the ICO pellet by centrifugation at 453 *g* for 5 min at 4°C.
      ***Note:*** Add ice-cold ADV+++ slow and dropwise to the thawed ICO to avoid any osmotic disruption of the fragile ICO due to fast environmental changes.
    iii. Resuspend the thawed ICO pellet into an appropriate volume of BME to seed the desired number of wells, well size, and number of 25 μL BME domes.
      ***Note:*** Basic knowledge and experience in culturing ICO is preferential to estimate the appropriate number of wells and size of the suspension well-plate upon thawing.
      ***Note:*** The level of debris, consisting of dead ICO fragments, could be as high as 50% directly after thawing (Figure 1D; black squared box with zoom in section). It is strongly advised to revive ICOs in a smaller culture environment as the original frozen number of wells within the cryovial (e.g., start in 1 well of a 48-well plate, when 1 well of a 24-well plate was frozen). The ICO require 3–5 days to fully recover, regaining original growth speed can take up to 2 weeks (Figures 1E and 1F; black squared box with zoom in section).
    iv. Solidify the seeded domes by incubating at 21°C–25°C for 3 min.
      ***Note:*** Prevent any movement of the plate during the three-minute 21°C–25°C incubation for the solidification of the BME domes, thereby preventing a shift and destruction of the BME dome.
    v. Flip the plate upside down in one smooth motion.
      ***Note:*** Flip the plate upside down in a smooth motion to ensure the distribution of the ICO through the complete BME dome. Otherwise, the ICO will sink towards the well-bottom within the BME dome, thereby limiting environmental growth space in 3D and potentially promoting well-bottom adhesion of the ICO towards a 2D outgrowth.
    vi. Incubate 45 min at 37°C.
    vii. Reverse the plate and add the appropriate volume of SEM to each seeded well depending on the well size.
      ***Note:*** When thawing cryopreserved established ICO, at least 10%–50% BME occupation of small (Ø 10–100 μm) ICOs should be visible directly after thawing or within 3–5 days of culture (Figures 1D and 1E; black squared box with zoom in section).
    viii. Switch to EM after 3 days, by removing the SEM and adding fresh 37°C pre-warmed EM (Figure 1E).
    ix. Every 2–3 days refresh the EM by removing the old media and adding fresh 37°C pre-warmed EM.
    x. Continue with the ICO maintenance and expansion protocol accordingly.
      ***Note:*** It is recommended to passage thawed ICO only when the culture reached 95% density in the BME dome after thawing (Figure 1F). Passaging the ICO at a lower density might reduce growth speed drastically, eventually requiring a longer recovering time. Since the overall density is relatively high, darkened, collapsed organoid structures might be visible at this point due to lack of space for structural outgrowth (Figure 1F; black squared box with zoom in section).
      ***Note:*** After thawing, a high variation in growth recovery can be observed between different donors. No clear donor-related characteristics or mechanistic pathways have been identified yet that might explain this phenomenon.
5. Expand ICO in EM for 2–3 weeks, until at least passage 3, not exceeding a maximum of passage 9.^1^^,^^2^^,^^4^^,^^7^
  a. Assess the cultures every 2–3 days for growth speed, density, unwanted bacterial, fungal or yeast growth, etc., and determine the desired mode of action.
    i. Remove EM and add fresh 37°C pre-warmed EM, when ICO cultures are occupying the BME dome for less than 80%, with an average size of Ø 10–500 μm, and without darkened color or thickened border or large extensive residual debris (Figure 1G; black squared box with zoom in section).
      ***Note:*** Some ICO could entrap cellular debris inside the lumen of the organoid structure itself, as a part of a passaging artifact or inner-membrane shedding. This phenomena will not hamper the successful viable outgrowth of the ICO culture, and is not a sign to passage ICO cultures prematurely (Figure 1G; black squared box with zoom in section).
    ii. ICO should be passaged when >80% of the BME is occupied, especially in the presence of large (Ø 800‒1,000 μm), and/or darkened or thickened border structures (Figure 1H; black squared box with zoom in section).
  b. When desired, prepare a viably frozen ICO stock batch at the earliest passage as possible.^1^^,^^2^^,^^4^^,^^7^
    i. Freeze the ICO cultures in Freezing Medium (FM) upon >80% dome density (Figure 1H), creating one frozen vial for each 2 wells of a 48-well suspension plate or 1 well of a 24- or 12-well suspension plate.
    ii. Organoids should be frozen in a small-middle size, ranging between Ø 10–500 μm.
      ***Note:*** When the overall size exceeds this range, break the organoids mechanically by vigorously pipetting 10–20 times up and down, prior to freezing.
    iii. Store the vials for 2–24 h in a CoolCell cryo-container at −80°C, following by long-term liquid nitrogen storage at −196°C.
      ***Alternatives:*** Besides CoolCell, similar type commercially available cryo-freezing containers could be used to serve as substitution, such as Mr. Frosty (Thermo Scientific; Cat#: 5100-0001).
  c. Start the step-by-step branching protocol once ICO cultures have reached >=passage 3 after initiation, or have been passaged twice after thawing, while maintaining a growth rate that requires passaging 1:2 at least once a week. In which the latter will confirm a log growth expansion. Do not use ICO lines that exceed 9 passages *in vitro.*
    ***Note:*** The ratio (1:2, 1:3 or 1:4) in which ICO need to be passaged upon 80% coverage (Figure 1H), might differ greatly between different ICO lines. Established culture experience with a specific ICO line should be used to estimate the correct splitting and seeding ratio. The overall rule of thumb is to rather overgrow the ICO culture than to split the line too soon. The latter will result in a rapid decrease in growth rate and loss of the culture.
    ***Note:*** If an established ICO line is thawed with a passage 3 or higher, it is strongly advised to at least passage the ICO two times (based on >80% density; Figure 1H) to enable complete recovery, before branching initiation is started.
    ***Note:*** Prevent bubbles upon pipetting BME. BME and alternative hydrogels, such as Matrigel, are viscous and difficult to pipet. However, bubbles will disrupt the matrix integrity and will induce premature hydrogel degradation. Consequently, the ICO require passaging earlier than necessary, which might result in the loss of the (too small) ICO structures (Ø <100 μm).
    **CRITICAL:** Always maintain BME at 4°C during the complete procedure. Thaw the required volume on ice approximately 30 min prior to use. BME vials can only be taken from ice when BME is added to the ICO pellet, after which the vial should be placed back on ice as soon as possible. Once BME has reached 21°C–25°C the matrix will solidify which is a close to irreversible process.

## Key resources table

**Table:** 

REAGENT or RESOURCE	SOURCE	IDENTIFIER
**Chemicals, peptides, and recombinant proteins**		

2-Phospho-L-ascorbic acid trisodium salt	Sigma	Cat#: 49,752-100G
A83-01	Cayman Chemical/Sanbio	Cat#: 9,001,799-25
Advanced DMEM/F-12	Gibco/Thermo Fisher Scientific	Cat#: 12,604,013
B27 Supplement without vitamin A	Gibco/Thermo Fisher Scientific	Cat#: 12,587,001
Bovine serum albumin (BSA)	Sigma-Aldrich	Cat#: A-7906-100G
Collagenase IA	Sigma-Aldrich	Cat#: C9891-100MG
Conditioned medium: Noggin	Home made	N/A
Conditioned medium: RSPO1	Home made	N/A
Conditioned medium: Wnt-3a	Home made	N/A
Cultrex PathClear Reduced Growth Factor BME	R&D Systems/Biotech	Cat#: 3533-010-02
Dexamethasone	Sigma-Aldrich	Cat#: D4902-100MG
DMEM	Gibco/Thermo Fisher Scientific	Cat#: 31966-021
DMSO	Sigma-Aldrich	Cat#: D8418-250ML
D-Phosphate buffered saline (D-PBS)	Life Technologies	Cat#: 14190169
EBSS	Thermo Fisher Scientific	Cat#: 24,010,043
EGF	GenScript Biotech	Cat#: Z00333-1
Ethanol 70%	Boom	Cat#: 80012461.5000
Fetal bovine serum	Gibco	Cat#: 10270-106
FGF-10	GenScript Biotech	Cat#: Z03314-1
Forskolin	Sanbio	Cat#: 11018-50
Gastrin I	Sigma-Aldrich	Cat#: G9145
Geneticin G418	Invivogen	Cat#: ant-gn-5
Glucose	Sigma	Cat#: G8270-1KG
Hepes	Lonza/Fisher Scientific	Cat#: BE17-737E
HGF	GenScript Biotech	Cat#: Z03229-1
ITS+ Premix	Life Technologies	Cat#: 41,400,045
UltraGlutamine	Lonza/Fisher Scientific	Cat#: BE17-605E/U1
N-2 Supplement	Gibco/Thermo Fisher Scientific	Cat#: 15,410,294
N-Acetyl-L-cysteine	Sigma-Aldrich	Cat#: A9165-25G
Nicotinamide	Sigma-Aldrich	Cat#: N0636-100G
Penicillin-streptomycin	Thermo Fisher Scientific	Cat#: 15,140,122
Primocin	InVivogen/Bioconnect	Cat#: Ant-pm-2
Recombinant human DKK1 protein	Abcam Ltd	Cat#: ab155623-100UG
Sodium pyruvate	Gibco/Life Technologies	Cat#: 11360-070
Sodium bicarbonate	Sigma	Cat#: S5761
Stemgent hES Cell Cloning and Recovery Supplement	Tebu-Bio	Cat#: 01-0014-500
TrypLE	Thermo Fisher Scientific	Cat#: 12,604,013
University of Wisconsin Solution	Bridge to Life (Europe) Ltd.	N/A
William-E Medium	Life Technologies	Cat#: 32551087
Y27632	MedChem Express/Bioconnect	Cat#: HY-10583_10mg
Zeocin	Invivogen	Cat#: ant-zn-1

**Experimental models: Cell lines**		

Human: Noggin cell line: 293T-HA-Noggin (stably transfected with pcDNA3 *NEO*, mouse Noggin, C-Terminal tag is Human IgG1-Fc)	Gift obtained from Hubrecht Institute, Utrecht, The Netherlands	N/A
Human: R-spondin cell line: 293T-H-RspoI-Fc	Gift obtained from Hubrecht Institute, Utrecht, The Netherlands	N/A
Human: Wnt-3a cell line: Wnt-3a L (stably transfected with pCDNA3.1Zeo-mouse Wnt-3a)	Gift obtained from Hubrecht Institute, Utrecht, The Netherlands	N/A

**Experimental models: Organisms/strains**		

Human: Healthy tissue-derived ICO line #1495	N//A	N/A
Human: Healthy tissue-derived ICO line #1520	N/A	N/A
Human: Healthy tissue-derived ICO line #1560	N/A	N/A
Human: Patient tissue-derived CCAO line #14	N/A	N/A
Human: Patient tissue-derived PCLDO line #1471	N/A	N/A
Human: Patient tissue-derived PCLDO line #1479	N/A	N/A

**Other**		

Corning suspension plates 48-well	Sarstedt	Cat#: 83,3923,500
Corning suspension plates 24-well	Sarstedt	Cat#: 83,3922,500
Corning suspension plates 12-well	Sarstedt	Cat#: 83,3921,500
Cryotubes	Merck Life Science	Cat#: V7634-500EA
Cryo-Freezing Container - CoolCell	Corning	Cat#: 432138
Pipettips P20	Sarstedt	Cat#: 70,3021
Pipettips P200	Sarstedt	Cat#: 70,3030
Pipettips P1000	Sarstedt	Cat#: 70,3050
T75	Greiner Bio	Cat#: 658170
T175	Sanbio	Cat#: 156205
0.22 μm filters – Large volumes (<100 mL and CM) Polyethersulfone membrane – Low protein binding	Merck Life Science NV	Cat#: S2GPU02RE
0.22 μm filters – Small volumes (>100 mL and organoid media) Polyethersulfone membrane – Low protein binding	Sarstedt	Cat#: 83.1826.001
15 mL tubes	No recommended vendor	N/A
50 mL tubes	No recommended vendor	N/A
Syringes	No recommended vendor	N/A
Serological pipets	No recommended vendor	N/A
Cell freezing container (Mr. Frosty)	No recommended vendor	N/A
Light/optical (inverted) microscope appropriate for cell culture with standard oculars, including at least 4× and 10× objective	No recommended vendor	N/A
EVOS Cell Imaging Systems – Digital Color Fluorescence Microscope	Life Technologies	AMEFC4300
Liquid Nitrogen tank	No recommended vendor	N/A

## Materials and equipment

### Preparation of required media components and stock solution aliquots

#### 0.1% BSA in D-PBS for stock preparation

Prepare 0.1% Bovine Serum Albumin (BSA) in sterile Dulbecco’s Phosphate Buffered Saline (D-PBS), by dissolving 50 mg BSA in 50 mL D-PBS. Filter the buffer through a 0.22 μm filter, and store at either 4°C for six months or long-term at −20°C up to one year.

#### 2-Phospho-L-ascorbic acid trisodium salt

Prepare a 0.2M stock solution by dissolving 1.98 g ascorbic acid in 50 mL sterile MQ and mix well to completely dissolve the solution. Filter the stock solution through a 0.22 μm filter, and store 10 mL aliquots at −20°C up to six months.

***Note:*** Preparation of the stock solution might be hampered due to difficulties in properly dissolving the powder. Pre-warming the sterile MQ up to 37°C prior to ascorbic acid addition will increase solubility.

#### A83-01 (selective inhibitor of TGF-βR1, ALK4 and ALK7)

Gently open the lid of the bottle to prevent any spill over from the powder, and add 11.8 mL sterile DMSO to 25 mg A83-01 to prepare a 5 mM stock solution. Mix by pipetting up and down multiple times to fully dissolve the complete stock solution. Store 500 μL aliquots long-term at −20°C for up to one year.

#### Basement Membrane Extract (BME)

Thaw the BME 24 h on ice within a cold room or fridge at 4°C. Mix the complete BME volume gently to prevent any air bubble formation. Aliquot the BME into 1 mL aliquots, while maintaining the BME on ice as much as possible. Directly store aliquots long-term at −20°C up to one year. Prior to use, thaw the required amount of BME on ice for approximately 30 min.

***Note:*** Only take BME out of the ice when it is added to the (BR)ICO pellet, and keep on ice as much as possible in between handling to prevent solidification at 21°C–25°C.

***Alternatives:*** BME can be substituted with a comparable extracellular matrix substrate, Matrigel (Corning; Cat#: 356231; Growth Factor Reduced Basement Membrane Matrix, Phenol Red-Free), while the procedure remains unchanged and obtains equivalent results.

#### Collagenase IA

Dissolve 100 mg of collagenase IA in 10 mL sterile EBSS, creating a 10 mg/mL stock solution. Mix by pipetting up and down multiple times to fully dissolve the complete stock solution. Store 1 mL aliquots long-term at −20°C for up to one year.

***Note:*** Aliquots should be frozen quickly and freeze-thaw cycles should be prevented, since this will damage the activity.

#### Dexamethasone

First prepare a 30 mM stock solution by dissolving 5.88 mg in 500 μL sterile DMSO and store 50 μL aliquots at 4°C for 30 days or −20°C for up to one year. Mix by pipetting up and down multiple times to fully dissolve the complete stock solution. Further dissolve this 30 mM stock solution 300× in sterile DMSO to obtain a 0.1 mM stock solution. Prepare 50 μL aliquots and store at 4°C for 30 days or long-term at −20°C for six months.

#### Epidermal growth factor (EGF)

Briefly centrifuge the vial prior to opening to spin down the contents into the bottom of the vial. Gently open the lid of the bottle to prevent any spill over from the powder. Dissolve 1 mg EGF into 20 mL 0.1% BSA/PBS to prepare a 50 μg/mL stock solution. Mix by pipetting up and down multiple times to fully dissolve the complete stock solution. Store 500 μL aliquots at 4°C for one week or long-term at −20°C for six months.

***Note:*** Aliquots should be frozen quickly and freeze-thaw cycles should be prevented, since this will damage the activity.

#### Fibroblast growth factor (FGF)-10

Briefly centrifuge the vial prior to opening to spin down the contents into the bottom of the vial. Gently open the lid of the bottle to prevent any spill over from the powder. Dissolve 1 mg FGF-10 into 10 mL 0.1% BSA/PBS to prepare a 100 μg/mL stock solution. Mix by pipetting up and down multiple times to fully dissolve the complete stock solution. Store 500 μL aliquots at 4°C for one week or long-term at −20°C for six months.

***Note:*** Aliquots should be frozen quickly and freeze-thaw cycles should be prevented, since this will damage the activity.

#### Forskolin

Gently open the lid of the bottle to prevent any spill over from the powder, and add 12 mL sterile DMSO to 50 mg Forskolin to prepare a 10 mM stock solution. Mix by pipetting up and down multiple times to fully dissolve the complete stock solution. Prepare 500 μL aliquots and store long-term at −20°C for up to four years.

**CRITICAL:** Harmful in contact with skin, prevent any contact by wearing protective gloves and clothing. Prevent any eye contact. If skin or eye contact has occurred, wash area with extensive amounts of water and soap, according to the institutional safety protocol.

#### Gastrin I

Add 4.7 mL D-PBS to 1 mg Gastrin I to prepare a 100 μM stock solution, and store 500 μL aliquots at −20°C up to three months. Mix by pipetting up and down multiple times to fully dissolve the complete stock solution. Further dissolve the 100 μM stock solution 10× in D-PBS to obtain a 10 μM stock solution. Store 500 μL aliquots at −20°C up to three months.

#### Glucose

Prepare a 1.4M stock solution by dissolving 12.6 g glucose in 50 mL sterile Milli-Q water. Mix by pipetting up and down multiple times to fully dissolve the complete stock solution. Filter the stock solution through a 0.22 μm filter, and store long-term at 4°C for up to one year.

***Note:*** Pre-warm the sterile Milli-Q water up to 25°C–37°C prior to glucose addition to increase solubility.

#### Hepatocyte growth factor (HGF)

Briefly centrifuge the vial prior to opening to spin down the contents into the bottom of the vial. Gently open the lid of the bottle to prevent any spill over from the powder. Dissolve 1 mg HGF into 20 mL 0.1% BSA/PBS to prepare a 50 μg/mL stock solution. Mix by pipetting up and down multiple times to fully dissolve the complete stock solution. Store 500 μL aliquots at 4°C for one week or long-term at −20°C for six months.

***Note:*** Aliquots should be frozen quickly and freeze-thaw cycles should be prevented, since this will damage the activity.

#### N-Acetyl-L-Cysteine

Dissolve 1.6 g of N-Acetyl-L-Cysteine into 20 mL sterile Milli-Q water by pipetting up and down multiple times, to obtain a 500 mM stock solution. Filter the stock solution through a 0.22 μm filter. Prepare 1 mL aliquots, and store at −20°C for up to one year.

***Note:*** The stock solution might be difficult to prepare due to insolubility of the powder. Pre-warming the sterile Milli-Q water up to 37°C prior to N-Acetyl-L-Cysteine addition will increase solubility.

**CRITICAL:** Prevent any eye contact to prevent any serious eye irritation. It is strongly advised to wash hands thoroughly after handling to hamper the transfer to the eyes. If powder/solution was in contact with the eyes, rinse with extensive amounts of water for several minutes. Gain medical attention when eye irritation persists.

#### Nicotinamide

Prepare a 1M nicotinamide stock solution by dissolving 12.2 g in 100 mL D-PBS. Mix by pipetting up and down multiple times to fully dissolve the complete stock solution and filter the stock solution through a 0.22 μm filter. Store 10 mL aliquots at −20°C for up to four months.

***Note:*** Aliquots should be frozen quickly and freeze-thaw cycles should be prevented, since this will damage the activity.

**CRITICAL:** Prevent any eye contact to prevent any serious eye irritation. It is strongly advised to wash hands thoroughly after handling to hamper the transfer to the eyes. If powder/solution was in contact with the eyes, rinse with extensive amounts of water for several minutes. Gain medical attention when eye irritation persists.

#### Recombinant human Dickkopf WNT signaling pathway Inhibitor (DKK)1 protein

Briefly centrifuge the vial prior to opening to spin down the contents into the bottom of the vial. Gently open the lid of the bottle to prevent any spill over from the powder. Add 2 mL 0.1% BSA/PBS to 100 μg DKK1 protein to obtain a 50 μg/mL stock solution. Mix by pipetting up and down multiple times to fully dissolve the complete stock solution. Aliquot into 200 μL vials and store at −20°C for six months.

***Note:*** Dissolve and aliquot DKK1 within two weeks of arrival, and avoid repetitive freeze-thaw cycles.

#### Sodium Bicarbonate

Dissolve 3.57 g Sodium Bicarbonate within 50 mL sterile MQ to obtain an 850 mM stock solution. Mix by pipetting up and down multiple times to fully dissolve the complete stock solution and filter the stock solution through a 0.22 μm filter. Store long-term at 4°C for up to one year.

#### Y-27632 dihydrochloride (Rock inhibitor)

Briefly centrifuge the vial prior to opening to spin down the contents into the bottom of the vial. Prepare a 10 mM Y-27632 stock solution by dissolving 10 mg within 3.122 mL sterile MQ. Mix by pipetting up and down multiple times to fully dissolve the complete stock solution. Aliquot into 100 μL vials and store long-term at -20°C for one month.

***Note:*** Aliquots should be frozen quickly and freeze-thaw cycles should be prevented, since this will damage the activity.

***Note:*** Protect stock solution from light as much as possible.

### Preparation of culture media

**Table:** Description and application of the different culture media types

Culture media	Abbreviation	Description	Application	Storage
Conditioned Media	CM	Dulbecco’s Modified Eagles Medium supplemented with 10% FBS, 1% UltraGlutamine, 1% Penicillin-Streptomycin and selection agents (described in table with CM recipe)	Preparation of conditioned culture media	8–12 weeks at 4°C
Wash buffer	ADV+++	Advanced Dulbecco’s Modified Eagles Medium with Nutrient Mixture F12 hams supplemented with 1% UltraGlutamine, 1% Hepes and 0.2% Primocin (concentrations described in table with ADV+++ recipe)	Handling ICO and preparation of culture media	8–12 weeks at 4°C
Start-Expansion Medium	SEM	ICO initiated culture medium supplemented with Wnt-3a CM, Noggin CM (both previously collected from producing cell line) and ROCK-inhibitor; Y-27632 (concentrations described in table with SEM recipe)	ICO medium used for the initiation of organoids	1 week at 4°C
Expansion Medium	EM	ICO expansion culture medium with canonical- Wnt-3a stimulation (concentrations described in table with EM recipe)	ICO medium used for the expansion of organoids	1.5 weeks at 4°C
Branching Medium	BM	BRCO branching medium with non-canonical- Wnt-3a stimulation (concentrations described in table with BM recipe)	BRCO medium used for the initiation of branching morphology	2 weeks at 4°C
Freezing Medium	FM	ADV+++ supplemented with 20% FBS and 10% DMSO	Freeze medium used for the viable frozen storage of ICO/BRCO	1 year at -20°C

**Table:** Conditioned Media (CM) for 293T-HA-Noggin, 293T-H-RspoI-Fc and Wnt-3a L

Reagent	Final concentration	Amount
DMEM	89%	500 mL
FBS	10%	50 mL
Penicillin-Streptomycin (10,000 U/mL)	1%	5 mL
UltraGlutamine (200 mM)	1%	5 mL
Selection:		
Noggin - Geneticin G418 (50 mg/mL)	500 μg/mL	5 mL
R-Spondin - Zeocin (100 mg/mL)	300 μg/mL	625 μL
Wnt-3a - Zeocin (100 mg/mL)	125 μg/mL	1.5 mL
**Total**	**NA**	**560 mL**

***Note:*** Prepare specific selection media for each cell line separately. Upon harvesting of the CM, use ADV+++ for the Noggin and R-spondin cell lines, and apply CM without selection agents for the harvest of the Wnt-3a cell line.

***Note:*** CM can be stored for 8–12 week at 4°C, after which fresh CM should be prepared.

**Table:** Wash buffer (ADV+++)

Reagent	Final concentration	Amount
ADV-DMEM/F12	98%	500 mL
Hepes (1 M)	1%	5 mL
Primocin (10,000 U/mL; 50 mg/mL)	0.2%; 100 μg/mL	1 mL
UltraGlutamine (200 mM)	1%	5 mL
**Total**	**NA**	**511 mL**

***Note:*** ADV+++ can be stored for 8–12 week at 4°C, after which fresh ADV+++ should be prepared.

**Table:** Start-Expansion Medium (SEM)

Reagent	Final concentration	Amount
ADV+++	41%	4.18 mL
A83-01 (5 mM)	5 μM	10 μL
B27 (50×)	2%	200 μL
EGF (50 μg/mL)	50 ng/mL	10 μL
FGF10 (100 μg/mL)	100 ng/mL	10 μL
Forskolin (10 mM)	10 μM	10 μL
Gastrin I (10 μM)	10 nM	10 μL
hES Cell Cloning and Recovery (1000×)	0.1%	10 μL
HGF (50 μg/mL)	25 ng/mL	5 μL
N2 (50×)	1%	100 μL
N-Acetyl-L-Cystein (500 mM)	1.25 mM	20 μL
Nicotinamide (1 M)	10 nM	100 μL
Noggin CM	25 ng/mL (10%)	1 mL
R-Spondin CM	1 mM (10%)	1 mL
Wnt-3a CM	0.3 nM (30%)	3.33 mL
Y-27632 (10 mM)	10 μM	10 μL
**Total**	**NA**	**10 mL**

***Note:*** SEM can be stored for 1 week at 4°C, after which fresh SEM should be prepared.

**Table:** Expansion Medium (EM)

Reagent	Final concentration	Amount
ADV+++	85%	85.25 mL
A83-01 (5 mM)	5 μM	100 μL
B27 (50×)	2%	2 mL
EGF (50 μg/mL)	50 ng/mL	100 μL
FGF10 (100 μg/mL)	100 ng/mL	100 μL
Forskolin (10 mM)	10 μM	100 μL
Gastrin I (10 μM)	10 nM	100 μL
HGF (50 μg/mL)	25 ng/mL	50 μL
N2 (50×)	1%	1 mL
N-Acetyl-L-Cystein (500 mM)	1.25 mM	200 μL
Nicotinamide (1 M)	10 mM	1 mL
R-Spondin CM	1 mM (10%)	10 mL
**Total**	**NA**	**100 mL**

***Note:*** EM can be stored for 1 week at 4°C, after which fresh EM should be prepared.

**Table:** Branching Medium (BM)

Reagent	Final concentration	Amount
2-Phospho-L-Ascorbic Acid Trisodium Salt (0.2 M)	0.2 mM	10 μL
Dexamethasone (0.1 mM)	0.1 μM	10 μL
DKK1 (50 μg/mL)	100 ng/mL	20 μL
EGF (50 μg/mL)	20 ng/mL	4 μL
Glucose (1.4 M)	14 mM	100 μL
Hepes (1 M)	20 mM	20 μL
ITS+ (100×)	1%	100 μL
Nicotinamide (1 M)	10 mM	100 μL
Penicillin-Streptomycin (10,000 U/mL; 50 mg/mL)	0.2%; 100 μg/mL	100 μL
R-Spondin CM	10%	1 mL
Sodium Bicarbonate (850 mM)	17 mM	200 μL
Sodium Pyruvate (100 mM)	6.3 mM	630 μL
UltraGlutamine (200 mM)	2 mM	100 μL
William-E Medium	76%	7.606 mL
**Total**	**NA**	**10 mL**

***Note:*** BM can be stored for 1 week at 4°C, after which fresh BM should be prepared.

**Table:** Freezing Medium (FM)

Reagent	Final concentration	Amount
Fetal Bovine Serum	20%	2 mL
DMSO	10%	1 mL
ADV+++	70%	7 mL
**Total**	**NA**	**10 mL**

***Note:*** FM can be stored for one year at −20°C, after which fresh FM should be prepared.

**CRITICAL:** Filter the prepared media through a 0.22 μm filter prior to use, excluding the ADV+++ used as washing buffer.

## Step-by-step method details

### Prerequisites for initiation of branching ICO culture

**Timing: 2 h**

This section of the protocol describes the preparations needed prior to ICO branching initiation.

1. Prepare a fresh batch of serum-free EM and BM as described in the EM and BM recipe table.
  a. Filter the media using a 0.22 μm filter.
  b. Store the media at 4°C for a maximum of one week, and pre-warm the media in a 37°C water bath prior to use for approximately 30 min.
2. Establish and maintain the *in vitro* ICO culture.
  a. Maintain an ICO culture within its log expansion growth phase, which has been passaged at least three times after tissue-derived initiation or at least two times after thawing.
  b. The ICO should be cultured in EM, and should not exceed passage number 9.

***Note:*** An ICO culture that has reached its log expansion growth phase requires passaging at least once a week with a 1:2 or 1:4 ratio, showing a >80% density one week after passaging (Figure 1H). A large variety in organoid sizes can be observed, ranging from Ø 10‒1,000 μm. In addition, the level of debris or dying single cell clumps should not exceed an approximate estimation of 10%.

***Note:*** The branching ability of an ICO line can only be determined after branching initiation has been completed. Unfortunately, 30% of ICO will not result in any branching structures and will maintain a cystic organoid growth. Previous research did not identify any underlying patient characteristics that could predict branching development, such as donor age, sex or liver disease, making selection of successful ICO lines beforehand impossible.^1^

### ICO branching initiation into BRCO

**Timing: 3 days**

This part of the procedure describes the initiation of BRCO culture in already established ICO cultures, which will result in the establishment of a mixture of both cystic and branching ICO structures which differs per ICO line.

**Figure 2:**
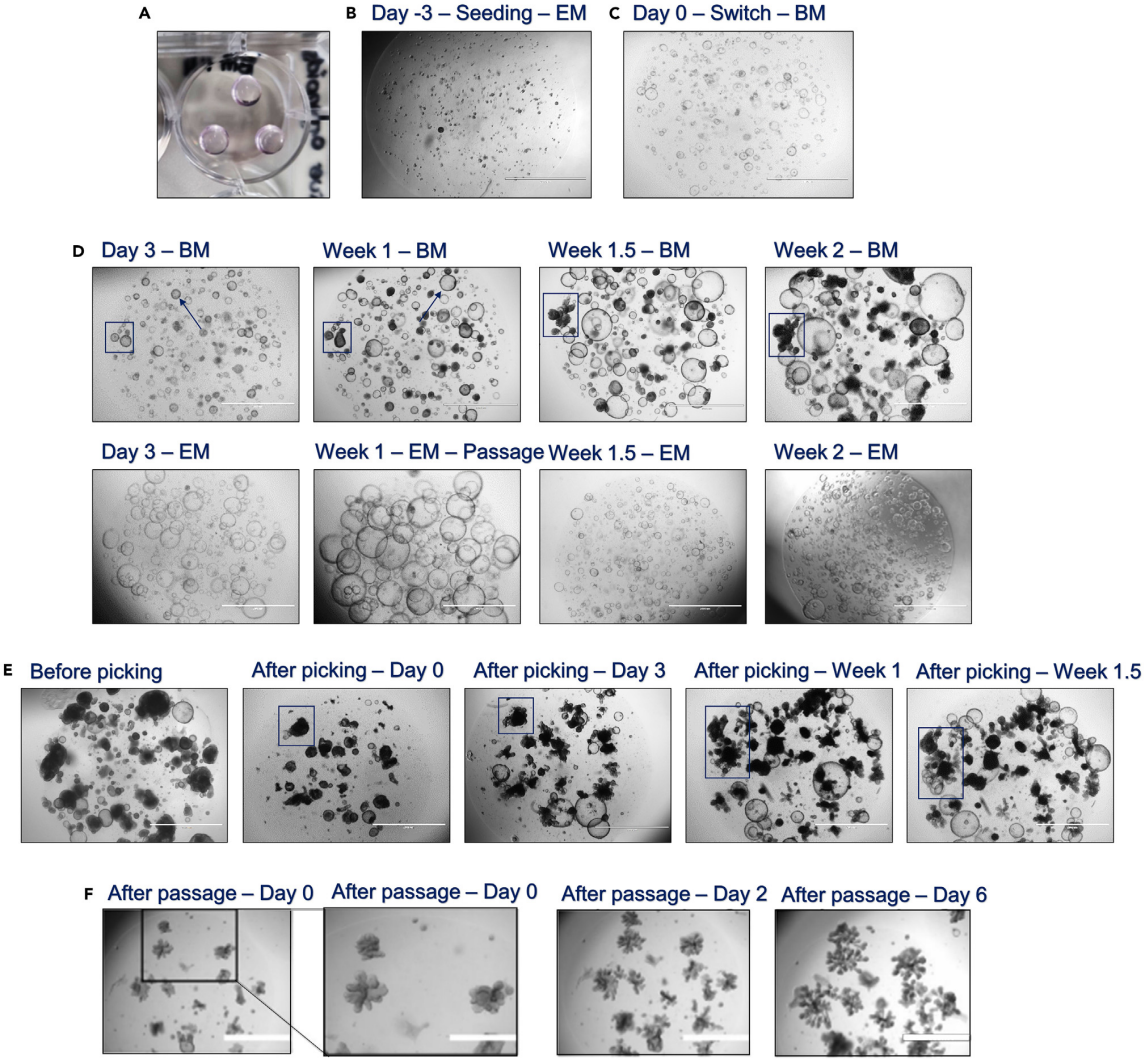
Bright field microscopic images of the branching initiation process in ICO cultures (A) The lay-out of the BME dome seeding in a well of a 12-wells plate upon branching initiation. (B) The desired (20%–50%) ICO seeding size and density to promote successful BRCO initiation. (C) After 3 days of EM and the presence of small ICOs (Ø 10–100 μm), the media is switched to BM. (D) BM incubation of 3 days will result in darkened ICO structures with a thickened border (blue arrow). The blue squared box tracks the branching formation of one of the BRCO over 2 weeks’ time. This specific ICO line already showed clear small tubular branching structures (Ø 100–500 μm) after 2 weeks of BM refreshment, while the EM control maintained exponential growth and required passaging after 1 week. (E) Tracking the outgrowth of branching structures until 1.5 weeks after manual BRCO clone selection, showing the formation of tubular structures after careful structure breaking during picking (blue squared box). (F) The 6 day outgrowth of BRCO after normal passaging procedures for the expansion of BRCO up to 1,000 μm (zoomed scale bar indicated 1,000 μm at 4× magnification), these results have been published as supplementary data in Roos et al., 2022; Supplementary figure S1^1^ (All images, except for A and zoom in: 2× magnification; scale bar indicates 2,000 μm).
  c. After 3 days, switch the EM by removing the old media and adding 1 mL fresh 37°C pre-warmed and filtered BM for one of the wells (Figure 2C). Continue culture with EM for the other well as a control of viable ICO growth after seeding.
    ***Note:*** The inclusion of the EM control will elucidate whether ICO culture would still maintain after seeding, or whether a technical error has occurred which could explain the lack of branching structures in advance (Figure 2D).

3. BRCO initiation.
  a. Maintain ADV+++ and BME at 4°C and pre-warm the filtered EM at 37°C.
    **CRITICAL:** Maintain ADV+++ at 4°C to enable collection of ICO from the BME. BME will turn to a more liquid state upon ice-cold temperatures, exceeding this temperature will result in BME solidifying during the process and greatly hampering successful ICO harvesting and seeding.
  b. Seed ICO at low density (20–50%) into two wells of a 12-well suspension plate, directly including a control well (Figure 2).
    i. Harvest 1 well of a 12-well suspension plate with a >80% dense ICO culture, ranging from Ø 10‒1,000 μm in individual organoid sizes (Figure 1H).
    ii. Remove culture medium from the well.
    iii. Add 900 μL ice-cold ADV+++ to the well, and collect the ICO by scraping the pipet tip across the bottom of the well and simultaneous resuspension.
    iv. Transfer the collected ICO into a 15 mL tube and add approximately 9 mL of ADV+++.
      ***Note:*** Directly apply the desired splitting ratio to ultimately obtain a 20%–50% seeding, by removing part of the ICO suspension (f.e. with a 1:6 splitting ratio, remove 7 mL of the ICO suspension and add an additional 7 mL of fresh ADV+++).
      ***Note:*** The ratio (1:3, 1:4, 1:6 or sometimes even 1:8) in which a culture needs to be passaged to reach a 20%–50% seeding density, might differ greatly between different ICO lines. Previous culture experience should be used to estimate the correct splitting and seeding ratio. Try to avoid making the organoids into a single cell suspension, as this will hamper the success rate of branching initiation tremendously. It is therefore not advised to count the cells in order to define the seeding density (refer to troubleshooting 2 or 3, when higher or lower seeding densities were applied).
    v. Diverse the ICO from the medium and remaining BME by centrifugation at 453 *g* for 5 min at 4°C.
    vi. Remove the supernatant without disturbing the ICO pellet.
    vii. Resuspend the ICO pellet in 200 μL ice-cold ADV+++.
    viii. Mechanically break the organoid structures into smaller fragments by vigorously resuspending, approximately 20 times using a P200 tip, against the bottom of the tube.
      ***Note:*** Upon breaking, organoids should maintain an approximate small-middle size range of Ø 10–500 μm. Based on the approximate size of harvesting, the force of resuspension should be determined. Elongate the resuspension time when organoid sizes exceed Ø >600 μm, to obtain the approximate size for branching initiation. Pay attention to avoid a single cell solution, and do not exceed resuspension times over 5 min, to prevent any harmful effects on the ICO culture.
    ix. Add an additional 2–3 mL ice-cold ADV+++ and collect the ICO by centrifugation at 453 *g* for 5 min at 4°C.
    x. Carefully remove supernatant, using either a vacuum system or pipetting, and dry the pellet as much as possible by utilizing a P200 to remove the residual ADV+++. Thereby further avoiding BME dilution in the next step.
      ***Note:*** Since ICO growth speeds rapidly decrease upon branching initiation, it is strongly advised to remove the supernatant as much as possible before adding the BME to the ICO pellet. This will result in a sturdy BME dome that will last the first few weeks of branching initiation. Consequently, preventing any unnecessary and untimely passaging of fragile branching structures due to degrading BME. In addition, ICO are more prone to initiate branching structures in a sturdier environment.
    xi. Carefully resuspend the dried pellet into 150 μL ice-cold BME to avoid bubbles.
    xii. Divide the ICO into 2 wells of a 12-well suspension plate by adding three 25 μL BME domes in each well in the shape of a triangle (Figure 2A).
      ***Note:*** To simplify the manual BRCO clone picking procedure in advance, seed the ICO into the corner wells of the 12-well plate.
    xiii. Incubate the plate for 3 min at 21°C–25°C, and flip the plate upside down in one smooth motion.
    xiv. Incubate the plate upside down for 45 min at 37°C.
      ***Note:*** Flip the plate upside down in a smooth motion to ensure the distribution of the ICO through the complete BME dome. Otherwise, the ICO will sink towards the well-bottom within the BME dome, thereby limiting environmental growth space in 3D and potentially promoting well-bottom adhesion of the ICO towards a 2D outgrowth.
    xv. Reverse the plate and add 1 mL pre-warmed EM for the first 3 days of culture (Figure 2B).
      ***Note:*** Gently add the EM against the sides of the well to prevent any disturbance of the BME domes.
      ***Note:*** Recovery of the ICO with EM after seeding is highly recommended to increase the success rate of the branching formation (Figures 2B and 2C).

### Maintenance of (starting) BRCO culture

**Timing: 3–8 weeks**

The estimated time for the formation of branching structures highly differs between different ICO lines. Some ICO lines might already show starting branching structures within one week after the BM switch, while other ICO lines might take up to one month before the first branches are forming. This section of the procedure will elaborate the protocol for the maintenance of initiated BRCO culture.

4. *In vitro* BRCO maintenance.
  a. Check BRCO for (starting) branching structures and refresh medium by removing the old media and adding 1 mL fresh pre-warmed 37°C BM every 2–3 days (Figure 2D).
    i. Culture the EM control as performed normally based on 80% density and organoid size (Ø 10‒1,000 μm), passaging the culture when required (see before you begin) (Figure 2D).
      ***Note:*** Although this part of the procedure might already take up to 3–4 weeks before the ICO initiated branching structures, the EM control ICO will restore its original growth speed, rapidly preceding the BM culture (Figure 2D). Consequently, the EM control needs to be passaged more frequently. Especially, since growth speed rapidly decreases upon culture in BM.
  b. Passage the BRCO when a density of >95% BME occupation is reached, or when the BME domes start to degrade and detach from the well bottom by applying a similar procedure as described for initial seeding (Figures 2E and 2F).
    ***Note:*** At the initiation stage within the procedure, a culture comprising solely of darkened cystic ICO could still be observed (Figure 2D; blue squared box). In some cases, only 2–3 tiny (Ø 10–100 μm) starting branching structures can be distinguished (Figure 2D; blue squared box). In which the cystic organoids will expand in size more rapidly compared to the branching structures. Passaging is only performed to enable a sturdier BME environment, to generate more space for structural outgrowth, or to break any cystic ICO with size exceeding Ø 500 μm. In addition, passaging of starting BCRO could be an option when original seeding was deemed too high (refer to troubleshooting 2 or 3). Once a pure BRCO has been established, passaging can be applied to further expand the BRCO culture for future experimental assays or viable stock freezing.
    i. Harvest the organoids by removing the culture media
    ii. Gently suspend the BME domes in 900 μL of ADV+++ while scraping across the bottom of the well.
      ***Note:*** Branching organoids can be broken into smaller fragments (Ø 10–500 μm), however it is strongly recommended to apply gentle resuspension techniques within the well plate. Subsequently breaking the larger sized cystic organoids (Ø 300‒1,000 μm), while maintaining the branching structures (Figure 2F).
    iii. Transfer the harvested organoids into a 15 mL tube and fill up the suspension towards 10 mL of ADV+++.
    iv. Centrifuge the organoid suspension at 453 *g* for 5 min at 4°C and remove supernatant.
      ***Note:*** Centrifuge the harvested organoids only once to prevent the loss and excessive breaking of any small branching structures.
    v. Dry the pellet as much as possible by applying a P200, removing any residual supernatant.
    vi. Resuspend the pellet in the appropriate amount of BME to passage the culture 1:1 or 1:2 by adding 75 μL or 150 μL respectively.
    vii. Prepare one or two wells of a 12-well suspension plate with each 3 domes of 25 μL.
    viii. Incubate the plate for 3 min at 21°C–25°C, and subsequently flip the plate upside down in one smooth motion.
    ix. Incubate the plate for 45 min at 37°C.
      ***Note:*** Flip the plate upside down in a smooth motion to ensure the distribution of the ICO through the complete BME dome. Otherwise, the ICO will sink towards the well-bottom within the BME dome, thereby limiting environmental growth space in 3D and potentially promoting well-bottom adhesion of the ICO towards a 2D outgrowth.
    x. Reverse the plate an add 1 mL of pre-warmed BM.
      ***Note:*** Gently add the BM against the sides of the well to prevent any disturbance of the BME domes.
    xi. Continue with checking the BRCO cultures every 2–3 days to determine the mode of action in refreshing medium for 1 mL 37°C pre-warmed BM or passaging.
      ***Note:*** Upon passaging, it can take up to 5–7 days before true branching structures are observed again. Unfortunately, some lines lose the ability to form branching structures after passaging, showing only cystic ICO after 2–3 weeks of culture (refer to troubleshooting 4). This shift in phenotype towards cystic organoids could be caused by technical difficulties during procedure handling. However, in some cases this shift presents due to yet uncovered underlying mechanisms. Currently, the specific rationale for this shift, either procedure handling or underlying mechanisms, is not distinguishable. Once again stressing the importance of a correct decision whether to passage or continue refreshing the medium. In this case, the rule of thumb would be to rather refresh the medium once too many, than to passage the line too early and risking the loss of potential branching structures.

### Manual BRCO clone picking (optional step)

**Timing: 2 h**

This part of the procedure is of special importance for initiated BRCO that maintain to present an elaborate mixture of both cystic ICO and small to middle sized BRCO (Ø 100–500 μm). The manual selection of these BRCO will not only purify the culture all together, yet it will also enable a more spatial environment to stimulate BRCO outgrowth into large, complicated branching structures of approximately Ø 2,000 μm. This section of the protocol will display a detailed description on the handling procedures for selective manual BRCO picking.

**Figure 3:**
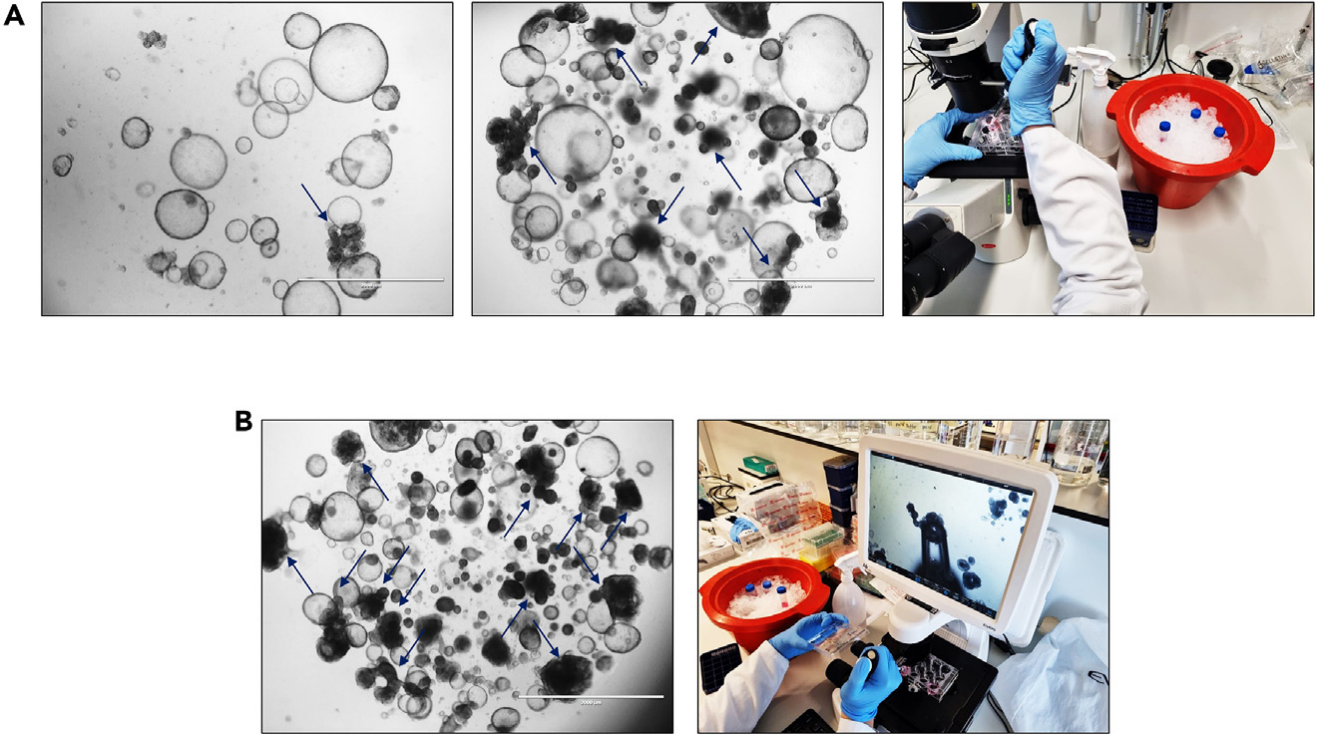
The manual clone selection of BRCO to enable the outgrowth of larger BRCO structures and the purification of the BRCO culture from cystic ICO structures (A) The BRCO density (<70%) without overlapping structures that could be picked by applying an inverted microscope, as indicated by the example in the picture. (B) The manual BRCO selection of high density BRCO cultures (>70%) with overlapping structures utilizing an automated hybrid microscope cell imaging system (EVOS Cell Imaging System, Thermo Fisher Scientific). The blue arrows indicate the BRCO structures that should be manually picked. (All bright field images; 2× magnification; scale bar indicates 2,000 μm).

***Note:*** This section of the protocol is only applicable for BRCO cultures that either include non-branching, cystic ICOs or contains multiple smaller BRCO (Figures 2E and 3). The manual selection of BRCO will enable a pure BRCO culture or the opportunity to promote outgrowth of larger BRCO. Some BRCO will develop a pure branching culture with large intrinsic tubular networks directly after initiation, these BRCO only require culture maintenance, as described in the section Maintenance of (starting) BRCO cultures.

***Note:*** Passaging of a pure BRCO culture might result in the recurrence of cystic ICO counterparts, meaning that some lines require multiple rounds of manual clone picking to restore a pure BRCO after every passage (refer to troubleshooting 4).

***Note:*** Somewhat degraded BME can simplify the picking procedure since it enables the piercing and separation of the BME more easily and therefore the selective collection of branching structure.

5. BRCO selection.
  a. Add 9 mL ice-cold ADV+++ into a 15 mL tube, and preserve on ice within an ice-bucket until required.
    ***Note:*** Keep this tube on ice during the complete procedure. None of the handling described requires to take the tube from the ice-bucket.
  b. Manual selection of BRCO is performed by picking the branching structures from the BME dome using a bright field microscope, leaving the cystic ICO behind in the BME dome. Two different techniques for manual selection can be applied, based on the overall density and spatial distribution of the culture of interest.
    i. Apply the correct selective BRCO picking strategy and continue the procedure accordingly.
  c. Manual BRCO clone picking with an inverted microscope upon lower quantity (<70%), non-overlapping, mixed BRCO cultures (Figure 3A).
    ***Note:*** When there is enough space between the cystic ICO and BRCO, without any overlapping structures (Figure 3A), clone picking can be performed by utilizing an inverted bright field microscope.
    i. Wipe all required materials, microscope and bench with 70% Ethanol, especially when working outside of the flow-cabinet.
    ii. Directly place the ice-bucket containing the 15 mL with ADV+++ next to the microscope system.
    iii. Do not remove the BM culture medium prior to manual picking.
    iv. Microscopically check the location of the desired BRCO clone for manual picking at the 4× and 10× magnification. Use the 10× magnification for further picking.
    v. Use a P200 or P1000, based on the preferential BRCO size and prevention of BRCO network breaking, to selectively pick the clone of interest (Figure 3A; blue arrows). Set pipets to 100 μL.
    vi. Take approximately 20–50 μL of ice-cold ADV+++ from the 15 mL tube, into the P200 tip. Keep fluids at the apex of the P200 tip.
    vii. Find the pipet tip underneath the microscope and bring in close proximity of the BRCO of interest.
    viii. Pierce the BME carefully and allocate the tip as close as possible to the BRCO of interest.
    ix. Gently eject the 20–50 μL into the BME, as close to the BRCO of interest as possible, to locally liquefy the BME.
    x. Gently suck the complete branching structure into the P200 tip, prevent the inclusion of any cystic ICO (refer to troubleshooting 5).
    xi. Transfer the BRCO into the 15 mL containing the ice-cold ADV+++, and resuspend approximately 5–7 times to remove the branching structure from the pipet tip (refer to troubleshooting 6).
    xii. Repeat cycle from step 5.C.VI until all BRCO of interest have been harvested (Figures 2F and 3A).
    xiii. Discard the plate containing the residual cystic ICO culture.
    xiv. Continue with step 5.E.
  d. Manual BRCO clone picking with the automated hybrid microscope cell imaging system (EVOS) upon high quantity (>70%), overlapping and mixed BRCO cultures (Figure 3B).
    ***Note:*** A highly dense culture of >70% BME occupation that contains overlapping structures of cystic ICO and BRCO or even clumped cystic ICO and BRCO structures, hampers selective clone picking without the inclusion of any cystic ICO. In this case, manual clone picking can be performed by using an automated hybrid microscope system (Figure 3B). Consequently, creating more space to manipulate the pipet upon picking and applying a lower magnification that can visualize the complete well all at once, which both benefit manual clone picking in high density cultures tremendously.
    i. Wipe all required materials, microscope and bench with 70% Ethanol, especially when working outside of the flow-cabinet.
    ii. Directly place the ice-bucket containing the 15 mL with ADV+++ next to the EVOS system.
    iii. Remove the BM culture medium prior to manual picking.
    iv. Add 1 mL of ice-cold ADV+++ from the 15 mL tube directly to the well of interest.
    v. Dissolve the BME domes by gentle resuspension and scraping as normally performed during passaging.
      ***Note:*** Make sure to only gently resuspend the BME domes, to prevent any unnecessary breaking of the branching structures.
    vi. Add an additional 1 mL of ice-cold ADV+++ from the 15 mL tube, and mix the BRCO suspension.
      ***Note:*** The BRCO suspension should increase the space among cystic and branching ICO structures, thereby reducing the amount of overlap.
    vii. Utilize the EVOS system to visualize the mixed BRCO suspension.
    viii. Selectively pick branching structures from the BRCO suspension using a P200 pipet and tip, prevent the inclusion of any cystic ICO (Figure 3B; blue arrows) (refer to troubleshooting 5).
    ix. Transfer any collect branching structures directly to the 15 mL tube, and resuspend approximately 5–7 times to remove the branching structure from the pipet tip (refer to troubleshooting 6).
    x. Repeat cycle from step 5.D.VIII until all BRCO of interest have been harvested (Figures 2F and 3B).
    xi. Discard the plate containing the residual cystic ICO culture.
    xii. Continue with step 5.E.
  e. Passage the manually picked BRCO clones obtained via inverted microscopy or the EVOS system, continuing with the 15 mL tube containing ice-cold ADV+++ and the selected BRCO.
    i. Centrifuge the manually picked BRCO suspension at 453 *g* for 5 min at 4°C.
    ii. Remove the supernatant, and dry the pellet as much as possible by removing any residual supernatant using a P200.
    iii. Add the desired amount of BME to the pellet, to constitute the same number of wells of a 12-well suspension plate as the picking procedure started with. Continue to apply three 25 μL domes per 12-well in a triangle shape (Figure 2A).
      ***Note:*** Apply a 1:1 passaging rate after manual BRCO clone picking, since this a stressful procedure and some branching structure might be lost during handling (Figure 2F).
    iv. Incubate the plate for 3 min at 21°C–25°C, and subsequently flip the plate upside down in one smooth motion.
    v. Incubate the plate for 45 min at 37°C.
      ***Note:*** Flip the plate upside down in a smooth motion to ensure the distribution of the ICO through the complete BME dome. Otherwise, the ICO will sink towards the well-bottom within the BME dome, thereby limiting environmental growth space in 3D and potentially promoting well-bottom adhesion of the ICO towards a 2D outgrowth.
    vi. Reverse the plate and add 1 mL of pre-warmed BM. Gently add the BM against the sides of the well to prevent any disturbance of the BME domes.
    vii. Continue with checking the BRCO cultures every 2–3 days to determine the mode of action in refreshing medium for 1 mL 37°C pre-warmed BM or passaging (Figures 2E and 2F).
      ***Note:*** The most optimal (sterile) setting would be to place a microscope in the flow-cabinet, preventing any potential bacterial and fungal contamination. However, when not available, manual BRCO clone selection can be performed outside of the flow-cabinet at the bench, after extensive cleaning with 70% Ethanol of all required materials. Make sure to keep an eye out for potential microbial contamination when using the latter option.
      ***Note:*** Wear gloves during the complete clone picking procedure (Figure 3).
      ***Note:*** Use sterile P200 pipet tips, even when working outside of the flow-cabinet, preventing any additional contamination sources. Do not utilize these tips within the flow-cabinet after non-sterile use. However, these tips can still be applied for non-sterile bench procedures.
      **CRITICAL:** To maintain a pure BRCO culture, a mixture of selective clonal picking and passaging needs to be applied.

### BRCO viable frozen storage and validation experiments

**Timing: Highly depends on desired experimental design**

**Figure 4:**
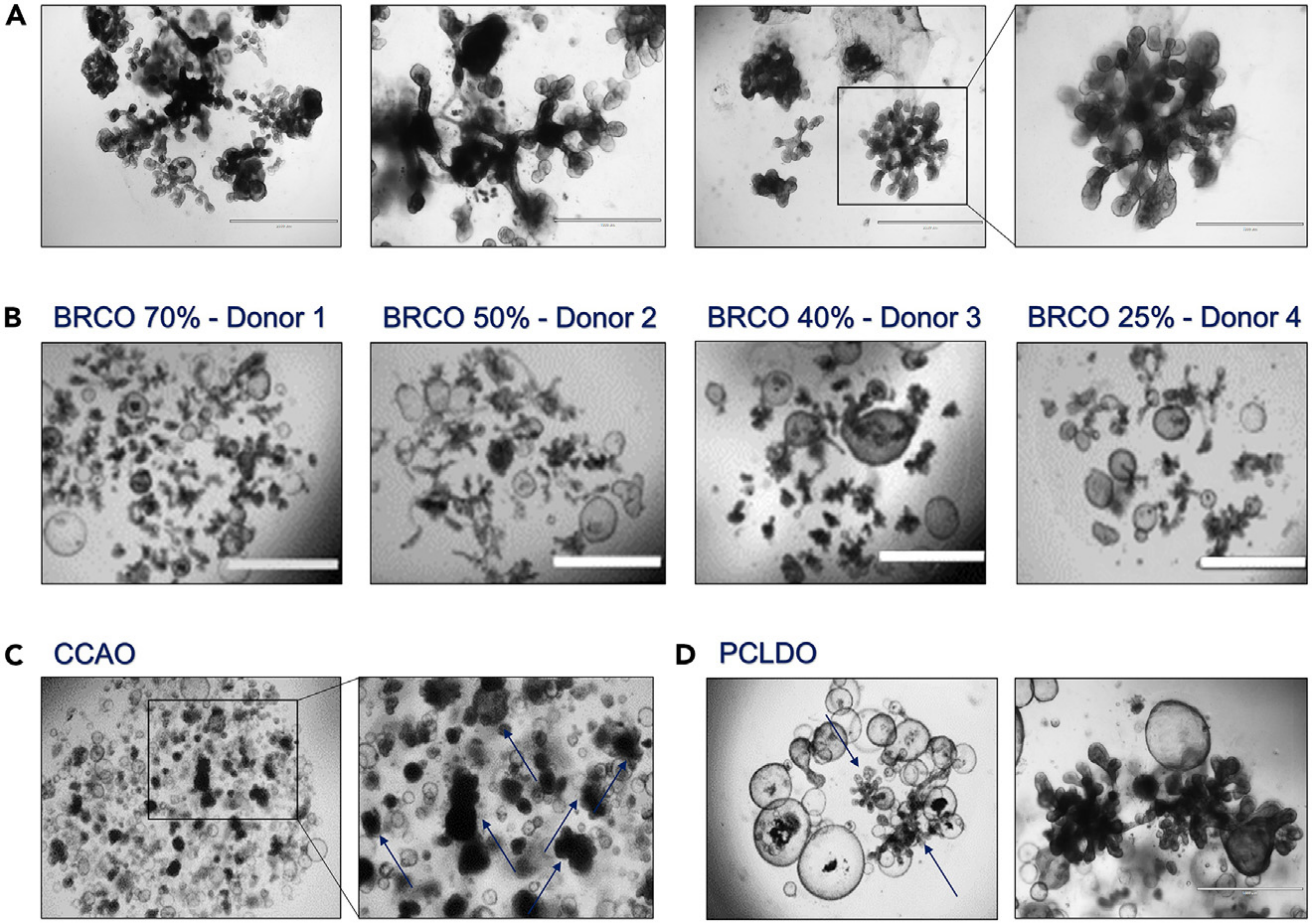
Bright field microscopic images of the expected outcomes of a successful BRCO initiation procedure (A) The formation of an elaborate tubular network of BRCO structures within one healthy ICO line (zoomed scale bar indicates 1,000 μm at 4× magnification). (B) Upon BM media switch, the percentage of BRCO outgrow differs between different healthy ICO lines, ranging from 70%‒25% of total organoid structures within the BME dome including different size ranges of (100–500 μm), these results have been published as supplementary data in Roos et al., 2022; Supplementary figure S1.^1^ (C) Successful branching of BRCCAO, showing the distinct dense branching structures without large elaborate networks or tubular formations reaching outwards (zoomed scale bar indicates 1,000 μm at 4× magnification). (D) The successful branching of PCLDO showing large elaborate tubular networks exceeding the 3,000 μm (zoomed scale bar indicates 1,000 μm at 4× magnification). (All images, except zoom in; 2× magnification; scale bar indicates 2,000 μm).

Once a pure BRCO culture has been established, the branching structures will reach extensive sizes with elaborate networks of tubular structures (Figures 2, 3, and 4). The maintenance and expansion of these BRCO lines highly depends on their future application in experimental assays and/or frozen storage. This section of the procedure elucidates the procedures for viable frozen storages and provides ideas for further validation experiments.

6. Viable frozen stock storage and validation experiments.
  a. Viable freezing of BRCO cultures is highly comparable to ICO viable freezing.
    i. Thaw a CoolCell cryo-container at 4°C for approximately 30 min, and maintain at 4°C until storage at −80°C.
    ii. Thaw frozen FM on ice for approximately 15 min, and maintain on ice during the procedure.
    iii. Print labels or pre-write the cryovials prior to starting the freezing procedure.
    iv. Harvest BRCO cultures by removing the culture medium, and gently resuspend the BME domes into 900 μL ice-cold ADV+++ by scraping the well bottom.
    v. Transfer the collected BRCO into a 15 mL tube and fill-up to 10 mL with ice-cold ADV+++.
    vi. Centrifuge the BRCO at 453 *g* for 5 min at 4°C.
    vii. Remove the supernatant and resuspend the pellet in 1 mL ice-cold FM with a 5 mL serological pipet and pipetboy combination.
    viii. Gently pipet a few times up and down until the pellet is completely dissolved, while small fragments are still visible within the suspension.
    ix. Add the required amount of FM to enable the freezing of one well of a 12-well suspension plate into one cryovial with a total volume of 1 mL respectively.
    x. Divide the BRCO FM into the required amount of cryovials, and store the cryovials into a 4°C CoolCell cryo-container by equally dividing the cryovials over the available locations within the cryo-container.
    xi. Within 5 min after FM addition, store the cryo-container containing the BRCO cryovials into a −80°C freezer for at least 2 h with a maximum of 24 h.
    xii. For long-term storage, transfer the cryovials to liquid nitrogen storage at −196°C, as soon as possible.
      ***Note:*** Since the BRCO pellet is not resuspended into BME, a completely dried pellet is not obligatory. Some residual supernatant is allowed upon resuspension into FM.
      ***Note:*** It is highly recommended to apply a 5 mL serological pipet rather than a P1000 upon pellet resuspension, to hamper any unnecessary breaking of the fragile branching structures upon viable freezing.
  b. Any further experimental assays highly depend on the research implementation of interest. BRCO lines should be expanded according to the described procedure until sufficient numbers for the further experimental assay have been reached.
    ***Note:*** Previous research of *Roos* et al. has extensively researched multiple applications for identification, differentiation, validation, and functionality experiments, such as: immunofluorescence whole mount imaging, time-lapse imaging, qRT-PCR, single-cell transcriptomic, Liquid Chromatography-Mass Spectrometry, FACS and branching tree network analysis, in both BRCO and BRCCAO.^1^ For an elaborate experimental design and procedures on assays, we refer to the extensive publication of *Roos* et al*.*^1^

## Expected outcomes

When this procedure is correctly applied, its most important outcome would be the successful establishment of viably expanding and self-organizing BRCO presenting extensive tubular networks and mimicking tubular formation (Figure 4A). However, the number of successful branching structures initiated within ICO, greatly differs among different patient tissue-derived lines (Figure 4B). By applying the aforementioned procedure, a pure BRCO line should be established with a minimum of cystic structures present.

The previous study of *Roos* et al. uncovered a close resemblance of BRCO formation and branching development.^1^ The branching organoid lines can be used for long-term (>20 passages) cultures without extra somatic mutations or chromosomal instability, and resemble a more mature cholangiocyte-like state while recapitulating the *in vivo* cholangiocyte heterogeneity.^1^ Although the specific underlying mechanisms of branching formation have yet to be uncovered, previous studies discussed an important key driver role for NOTCH signaling. The importance of NOTCH signaling was demonstrated by its role in BRCO reversibility into cystic ICO cultures upon inhibition of its function. In addition, a clear resemblance was found in tip-driven ductal growth and spatial organization, indicating that BRCO show similar tubular formation as *in vivo* bile duct formation. However, BRCO comprise of a mature cholangiocyte cell type only, and do not represent a broad spectrum of different liver cell types as shown by transcriptional analysis at the single cell level. Further research is currently ongoing to determine its use in co-culture systems representative to the *in vivo* liver, organ-on-a-chip models and disease model drug-responses.

Branching organoids are revolutionizing the use of *in vitro* models for functional bile duct studies, branching development and a broad spectrum of different biliary diseases. In addition to creating BRCO, this procedure also successfully created branching structures in organoids initiated from biliary diseases, such as bile duct cholangiocarcinoma (BRCCAO) and PCLDO (Figures 4C and 4D; blue arrows). Further detailed analysis is currently ongoing. Of note, BRCCAO maintain their original mutational background, transcriptomic signature and exhibit a more representative *in vitro* tumor architecture and transcriptome that benefits current disease research and drug-response studies.^1^ BRCCAO drug responses presented a more comparative sensitivity similar to *in vivo* CCA responses. Further research should be performed to elucidate the complete capabilities of this branching protocol in other biliary disease models and even other types of hepato-pancreato-biliary (HPB) organoids.

A broad range of different experimental assays could be applied for further BRCO studies, and to better understand unknown mechanisms. Both immunofluorescence and qPCR assays can be applied to measure the expression level of important cholangiocyte markers, such as increasing KRT19 and decreasing KRT7 expression, hepatocyte markers, such as higher SERPINA1 and Albumin expression levels, and progenitor markers, such as limited SOX2 and LGR5 expression.^1^ Validation of the opened branching lumen and mapping of the connected tubular structures could be performed by active transport of fluorescent rhodamine-123 via Multi Drug Resistance (MDR).^1^

## Limitations

The correct use of this protocol would induce the BRCO initiation and expansion in at least 70% of the included ICO cultures. Nevertheless, 30% of the ICO undergoing branching initiation will not show any phenotypic chances in structural behaviors, even after 2 months of culture. Success rates highly depend on the ICO size and density distribution upon the shift from regular to branching culture medium. If ICO are seeded too small within too low densities (<20%), culture viability will be compromised and ICO expansion or branching will be severely hampered. Although extensive research has been performed on the identification of potential underlying patient characteristics that could play an important role in determining BRCO success rates beforehand, no prognostic patient-specific factors, such as age, sex or disease type, have been identified to be statistical relevant.^1^ This is deemed a major limitation in the application of the procedure, since successful BRCO initiation can only be deemed a failure after 1–2 months of culture and therefore asking a lot of patience and time. Importantly, extensive research showed that extrahepatic cholangiocyte organoids and fetal liver-derived organoids lack the ability to form these intrinsic branching structures. Taken together, BRCO initiation can only be achieved in adult- intrahepatic tissue-derived cholangiocyte organoids that have been passaged as ‘regular’ ICO for at least three passages.

These same restrictions are observed for BRCCAO that can only be initiated in already established CCAO and not directly from tumor biopsies. Although the exact mechanism is not yet clear, the switch to branching morphology seems only be feasible through a shift from the original organoid medium to specific branching medium. The mechanistic pathways behind this phenomenon are currently being addressed.

Comparative differences in growth speed, phenotypic behavior and survival in both ICO and BRCO could be appointed to donor age or sex variation. These differences greatly affect the use in comparative analysis, stressing the importance of ICO or BRCO selection to reduce donor variation influences as much as possible. Nevertheless, these ICO and BRCO cultures reflect the differences between (donor) individuals, making these models potentially very suitable for personalized medicine applications.

The procedure to initiate branching structures in otherwise spherical 3-dimensional cultures, allows for unprecedented disease modeling. However, the manual clone picking promotes a clonal outgrowth of a specific subset of branching structures, hampering heterogenetic outgrowth and biological replicates. In addition, potential variations in self-produced media components, such as R-spondin, Noggin and Wnt-3a, could have a major influence on ICO and/or BRCO quality. Consequently, it is highly recommended to include multiple different ICO lines based on age and sex, to obtain an average panel and a suitable comparative model.

**Figure 5:**
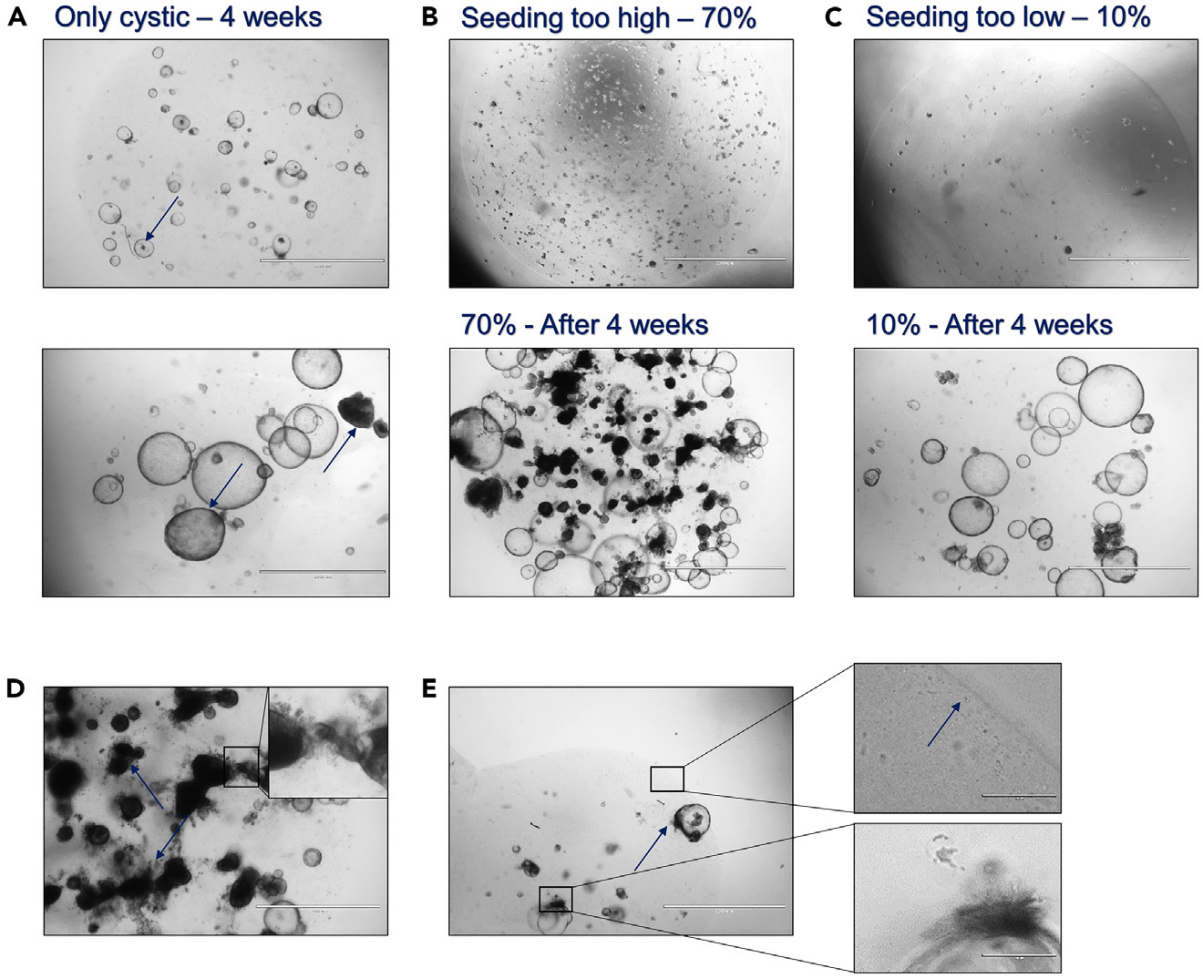
An overview of bright field microscopic images presenting potential problems that could arise during the BRCO initiation procedure (A) The presence of large cystic ICO structures (Ø 1,000 μm) only, even after 4 weeks of BM culture. The blue arrows indicate the somewhat thickened border and darkened coloring of the cystic organoids after 4 weeks of BM culture. (B) Exceeding the advised ICO seeding density >70% for BRCO initiation will result in the formation of smaller, dense and seemingly less viable BRCO structure, creating an overall less successful BRCO culture. (C) While decreasing the ICO seeding to <20% could result in the outgrowth of faster growing cystic ICO cultures, with only 1 or 2 BRCO that would be eligible for manual clone selection (increasing overall procedure times drastically). (D) When BRCO are not broken or passaged on time, the BRCO structure will start to expire over time showing very dark dense thickened tubular structures. The zoomed in black square indicates the release of dying cells into the BME dome. (E) Mycoplasma contamination of an ICO culture showing the distinct bearded ICO structures and the small dots (Ø 1–5 μm) that are both clear indicators for mycoplasma contamination (20× magnification; scale bar indicates 200 μm) (All images, except zoom in; 2× magnification; scale bar indicates 2,000 μm).

Initiated BRCO are very fragile structures and are highly affected by handling and environmental chances. The *in vitro* expansion of BRCO requires quite some patience, since growth speeds rapidly decreases. As a consequence, the acquirement of sufficient numbers of BRCO to perform (high-throughput) experimental assays could take up to 3 months. While BRCO could fail to resemble any branching structures after manual clone picking or passing for expansion, by reversing to their normal non-branching cystic ICO state that could quickly outgrow their branching counterpart (Figure 5A).

## Troubleshooting

### Problem 1

No branching structures formed after 6–8 weeks of BM initiation, while correctly applying the described procedure (Figure 5A).

### Potential solution

Approximately 30% of the ICO cultures will not initiate branching structures after weeks of culture, presenting cystic structures with somewhat thickened lineage or dark dense structures with dying cells. Till this date, the underlying reasons, such as age, sex, or molecular background, have yet to be uncovered. In addition, the success rate of other organoids derived from the HPB lineage, such as ductal pancreas and pancreatic ductal adenocarcinoma, has yet to be determined.

• Make sure the passage number of the ICO is at least 3 and does not exceed 9.
• It is advised to passage the line once more using the aforementioned procedure. If no branching structures form after a second round, deem the ICO line as a non-branching line and discard the plate.
• Double check whether the origin of the tissue location was not extrahepatic, or derived from fetal liver, since organoids initiated from these subsets are not capable to form any branching morphologies.

### Problem 2

The starting density of the ICO seeding for BRCO initiation is too high (Figure 5B) (related to step 3B).

### Potential solution

Harvest the seeded ICO again, and dilute the suspension accordingly to obtain a 20%–50% seeding of ICO prior to BRCO initiation. A lower seeding density simplifies manual picking of branching structures immensely, while preventing a reduction of BME stiffness or cystic ICO overgrowth.

### Problem 3

The starting density of the ICO for BRCO initiation is too low (Figure 5C) (related to step 3B).

### Potential solution

Seeding an even lower density than 20% could hamper the outgrowth of any organoid structures at all. Both cystic ICO and BRCO structures require a certain support from neighboring organoid structures. It is strongly advised to repeat the ICO seeding for BRCO initiation. If the EM control revived the low seeding densities, then this condition could be used to re-seed the organoid line. Otherwise a new viable frozen stock vial should be thawed to restart the complete procedure from ICO initiation.

• If the problem is noticed directly after seeding, it might benefit to culture the organoids in EM until at least an ICO occupation of 40%–50% is reached within the BME dome prior to switching to BM medium once again.

### Problem 4

BRCO structures disappeared completely after passaging, clone picking, or thawing (Figure 5A) (related to step 4B).

### Potential solution

Branching structures are very fragile, therefore breaking the BRCO too harshly into too small fragments could hamper the branching outgrowth. It could take up to 6 weeks before small fragmented BRCO are reforming intricate networks once again. Unfortunately, in some cases the branching ability is lost, resulting in a cystic ICO culture. The only available solution would be to restart the procedure from the beginning including the specific ICO line of interest. After performing the complete procedure again, the branching abilities should be initiated once again.

### Problem 5

During manual BRCO picking, a cystic ICO has been collected (related to steps 5C-D).

### Potential solution

If the cystic ICO is observed while entering the pipet-tip, the ICO can be removed by ejecting the ICO outside of the BME dome in the surrounding medium. When there was already a BRCO collected, the BRCO can be re-obtained from the surrounding medium and transferred into the 15 mL tube.

If the ICO is observed after picking and seeded in BME afterwards, the procedure with BM culture should be continued. The cystic ICO will either induce BRCO or can be removed by a second manual clone picking round when ICO have reached the spatial occupation of the BME and branching structures have re-formed.

### Problem 6

After manual BRCO clone picking, no pellet is obtained or no organoid structures are visible after seeding (related to steps 5C-D).

### Potential solution

ICO and BRCO are known to stick to plastic materials, such as 15 mL tubes or pipet tips. In some cases, this could drastically hamper the harvest of BRCO during manual clone picking. To prevent any losses, applied pipet tips and tubes could be coated with 0.1% BSA/PBS prior to use within the procedure. Resuspending the tips and swirling the tubes with 0.1% BSA/PBS for 1 min would already be sufficient to prevent any BRCO or ICO sticking to the plastic materials.

### Problem 7

Established BRCO structures are dying (Figure 5D).

### Potential solution

BRCO structures will not survive an outgrown BME density, highly decreased BME stiffness, or too large network formations. All will hamper correct growth factor depositions towards the BRCO structures. Upon direct handling when dying structures are visible, the BRCO culture could be saved. Either passing the BRCO line completely by expansion, or manual clone picking and tender BRCO breaking could induce cell viability and enable the outgrowth of viable BRCO structures once again. However, when no action is taken, the BRCO line could be lost due to the high numbers of cell death and the lack of viable cells to support BRCO outgrowth. In this case, the BRCO initiation procedure should be repeated with the thawed ICO line of interest.

### Problem 8

A bacterial, mycoplasma, fungal or yeast infection occurred after non-sterile manual BRCO clone picking.

### Potential solution

Upon bacterial contamination, either a clear halo will be visible surrounding the BME dome, very dark small dense (Ø 20–50 μm) rounded balls within the BME, or very tiny (Ø 1–5 μm) transparent dots at the outer brim of the BME dome can be observed when the culture is contaminated. While a mycoplasma infection will result in organoids presenting beard like structures with darkened borders (Figure 5E; zoomed square). In addition, very tiny dots (Ø 1–5 μm) can be observed within the complete BME dome (Figure 5E; blue arrow). On the other hand, fungal contamination will result in its distinct thin branching structures, while yeast will present the well-known beaded chain. Besides visual marks, a clear difference in ICO or BRCO culture behavior can be observed. Growth speed will rapidly decrease maintaining only some viable cells without any exponential outgrowth. In addition, the organoid structures will be starting to die and present darkened spots or cellular debris surrounding its structure. Unfortunately, once a contamination has infected the culture, no real solutions are available to cure the contamination from the BRCO line. The procedure to initiate BRCO cultures should be repeated with the thawed ICO line of interest.

• If contamination arose after manual clone picking, it is advised to clean the bench, microscopes and surrounding areas properly with ethanol, or to place the microscope into the flow-cabinet to create a more sterile environment upon the next manual clone picking.

## Resource availability

### Lead contact

Further information and requests for resources and reagents should be directed to and will be fulfilled by the lead contact, Monique Verstegen (m.verstegen@erasmusmc.nl).

### Materials availability

This study did not generate new unique reagents.

## Data Availability

No new codes have been generated.
